# A low-altitude public air route network for UAV management constructed by global subdivision grids

**DOI:** 10.1371/journal.pone.0249680

**Published:** 2021-04-14

**Authors:** Weixin Zhai, Bing Han, Dong Li, Jiexiong Duan, Chengqi Cheng

**Affiliations:** 1 College of Information and Electrical Engineering, China Agricultural University, Beijing, China; 2 Academy for Advanced Interdisciplinary Studies, Peking University, Beijing, China; 3 School of Earth and Space Sciences, Peking University, Beijing, China; 4 College of Engineering, Peking University, Beijing, China; Fuzhou University, CHINA

## Abstract

With an increasing number of unmanned aerial vehicles (UAVs), the difficulty of UAV management becomes more challenging, especially for low-altitude airspace due to complicated issues of security, privacy and flexibility. Existing management approaches to UAV flights include implementing registration of flight activity for supervision purposes, limiting the maximum flight height, setting different zones for different flight activities and prohibiting flights. In this research, we proposed a new air traffic management method for UAVs based on global subdivision theory. We designed four types of low-altitude air routes from grids, which correspond to grid sizes of 1.85 km, 128 m, 64 m and 32 m. Utilization of the subdivision grids transforms the complex spatial computation problem into a query process in the spatial database, which provides a new approach to UAV management in the fifth-generation (5G) era. We compared the number and data size of stored track records using longitude and latitude and different grid levels, computed time consumption for air route trafficability and simulated UAV flight to verify the feasibility of constructing this type of air traffic highway system. The amount of data storage and time consumption for air route trafficability can be substantially reduced by subdivision. For example, the data size using traditional expressions of latitude and longitude is approximately 1.5 times that of using a 21-level grid, and the time consumption by coordinates is approximately 1.5 times that of subdivision grids at level 21. The results of the simulated experiments indicate that in the 5G environment, gridded airspace can effectively improve the efficiency of UAV trajectory planning and reduce the size of information storage in the airspace environment. Therefore, given the increasing number of UAVs in the future, gridded highways have the potential to provide a foundation for various UAV applications.

## Introduction

Over the last several decades, researchers and manufacturers have shown increasing interest in utilizing unmanned aerial vehicles (UAVs) for diverse purposes. UAVs are widely utilized in various domains, such as agricultural applications [1], environmental and wildlife monitoring [2], surveillance [3], public safety [4–6], photogrammetry for three-dimensional (3D) modeling [7, 8], and logistics [9]. UAVs boast many advantages, including high maneuverability, low energy consumption, and the capacity for real-time monitoring.

Low-altitude UAVs, which is a common type of UAVs, have the potential to be extensively employed in civil aviation for different applications and purposes. The low-altitude airspace is defined by three specific requirements [10–12]: it is low enough to be affected by ground facilities and natural geomorphology, low enough to be covered by a ground mobile communication signal, and low enough to be separated from traditional general aviation and civil aviation activities. Statistics of the Civil Aviation Administration of China in 2018 demonstrated that the scope of the activity space for more than 80% of “low, slow and small” UAVs is below 120 m [13]. However, despite the economic potential of these vehicles, the management of low-altitude airspace is an unsolved and complicated problem that involves issues of security, privacy and flexibility. The technologies and policies that have proven applicable in medium- and high-altitude airspace cannot be applied to low-altitude airspace due to differences in factors such as the underlying surface, communication capability, and aircraft density.

Currently, the management of low-altitude UAVs in different countries is categorized into three groups. First, the registration of flight activity is implemented for purposes of supervision. Drone pilots are required to submit their real names and other information when applying to perform flight activities [14–16]. Second, the maximum flight height is limited for different types of UAVs. For example, the Federal Aviation Administration (FAA) restricts UAV pilots from accessing controlled airspace at or below 400 feet [14]. In Europe, the low-altitude airspace generally extends to at least 500 feet above the ground [17], and in China, the flight altitude of small civil UAVs is limited to 120 m [13]. Third, different zones are set for different flight activities or where flights are prohibited. Typically, civilian UAVs are not allowed near people, buildings, military zones, airports and other sensitive areas [18]. In the United Kingdom, recreational/commercial UAVs that weigh 20 kg or less must fly more than 30 m away from people and more than 50 m away from buildings [19]. Xu et al. comprehensively reviewed the regulatory policies and technologies of low-altitude UAV management [20]. Generally, although UAV airspace control measures adopted by different countries are effective, they are relatively simple and unsuitable for future scenarios with an increasing number of UAVs. In addition, with the breakthrough of 5G technology, the communication field [21, 22] has been revolutionized. The communication ability of UAVs also has been further strengthened, which provides further strong support for the management of UAVs in low-altitude airspace. Recently, researchers have made progress in the construction of a public air route network for UAVs that may contribute to UAV management at low altitudes [20].

Referring to the idea of discrete global grid systems (DGGSs) in this paper, we adopted a geographical coordinate subdividing grid with one-dimensional integer coding on a 2n tree in a three-dimensional (GeoSOT-3D) system to construct a low-altitude air route to address the UAV management issue in low-altitude airspace. First, we established a unified spatial grid subdivision system for the investigated airspace. Second, we mapped the spatial and temporal information of the UAVs and other spatial entities onto different grids. Last, the results indicated that the multilevel massive data query engine supported the implementation of multiple processes with high concurrency and massive amounts of data. The proposed method is an innovative and feasible approach to efficiently manage the UAVs in low-altitude airspace. The public air route network is constructed by regular 3D grids, instead of the previous management based on 3D coordinates. The advantage of the low-altitude public air route network is especially obvious in the case of large-quantity and high-speed computing requirements. The network also maximizes airspace utilization and adapts to the 5G communication environment while ensuring security.

## Literature review on management of low-altitude UAVs

In contrast to high-altitude airspace, where few obstacles exist, flights in low-altitude airspace are constrained by complicated environmental factors, such as terrain features, buildings, extreme weather conditions and human factors, such as safety and confidentiality requirements.

Due to the complexity of UAV operation, it is necessary to determine the operational roles of operators and service providers, and staff must be well trained. Therefore, management systems must be examined to reduce unnecessary human errors and to improve the efficiency of operation. Emergencies such as link loss, communication loss or loss of control are additional important factors in the management of low-altitude airspace for UAVs.

Two recognized UAV management projects exist in practice. The Metropolis project proposed four airspace designs in the urban airspace of a city, the size of Paris, with a projected population of 14 million by 2050 [23]. The four airspace concepts ranged from a decentralized direct routing concept to a highly structured tube network using four-dimensional (4D) trajectory-based operations [24]. The experimental results of the project demonstrated that extreme traffic densities can be achieved by spreading traffic over the airspace while keeping the structure relatively flexible. Singapore’s Traffic Management of Unmanned Aircraft Systems (TM-UAS) program proposed incorporating a structured airspace design into the existing urban infrastructure to ensure efficient and safe UAS operations [25]. Safe and efficient operations can be achieved by describing the urban airspace in terms of dynamic air blocks, with communication, navigation and surveillance capabilities that are potentially being assessed within these air blocks to define whether it is safe to operate across them. This work showed the possibility of reducing the number of conflicts via better airspace management and UAS control.

Geofences, a widely applied concept that defines virtual boundaries in a specific geographical area to ensure safe separation, have recently attracted interest in the research community [26–28]. Once the defined boundaries are violated, the UAV pilot will be informed, and the power will be cut off in some cases [29]. Nevertheless, as a conservative and uniform flight restriction to provide protection from UAVs, geofences restrict the exploitation of low-altitude airspace. The current research and established regulations on geofences are still incomplete [30, 31]. Researchers have proposed several theoretical models and techniques for applications in different scenarios. Bulusu et al. showed that cooperation among aircraft greatly improves UAS traffic volumes with simulations in the US San Francisco Bay Area and Norrköping, Sweden [25]. Lundberg et al. addressed challenges of designing future unmanned air traffic management concepts by integrating work domain analysis with conceptual design [32]. Motlagh et al. [33] presented a comprehensive survey and highlighted the potential for the delivery of low-altitude UAV-based Internet of Things (IoT) services from the air.

The UAV low-altitude public air route network proposed by Liao [20] of China is a novel solution for UAV regulation in low-altitude airspace that has attracted widespread attention from researchers, policy makers and the public. The UAV low-altitude public air route network is a set of air corridors of certain widths in a separate airspace for UAVs below the minimum flight height of manned aircraft. The air route network can be categorized into four types according to size, terminal point locations and functions: backbone route, trunk route, feeder route and terminal route. Experiments were conducted in Tianjin, China to standardize the order of low-altitude traffic, improve the utilization of low-altitude airspace resources and maintain aviation and public safety [12].

## Methodology

The existing method of constructing a low-altitude public air route network for UAVs is based on corridors with different diameters and entails a minimum distance between two air routes. Although the existing method has proven its capability of ensuring the separation of UAVs and civil aviation and maintaining flight safety, its adaptability to a complex environment and efficiency in the case of a massive number of UAVs needs to be further examined. Air routes should be added or altered according to changes in the underlying surface conditions and natural conditions; however, operation in a complex environment is time-consuming, especially in emergency cases. The computations of spatial relationships between air routes and various spatial fields are complicated and involve the solution of multiple nonlinear equations. Therefore, it is essential to advance an improved UAV low-altitude public air route network construction method to adapt to various requirements.

Recent studies on DGGSs have proven their advantages in UAV applications [34, 35]. Gridding, as a promising and important technology, was incorporated into the standardization roadmap for unmanned aircraft systems by the American National Standards Institute (ANSI) Unmanned Aircraft Systems Standardization Collaborative (UASSC) in June 2020. Bangkui Fan et al. argued that the unified management of spatial information on the UAV platform can be effectively realized by adopting the spatial grid model represented by GeoSOT-3D, because it will reduce the workload of flight management, airborne environmental perception, and range in the neighborhood by the association and queries of all spatial grid data [36]. The proposed algorithm based on DGGSs can perform real-time (dynamic/static) conflict detection on both individual aircraft and aircraft flying in formations with more efficient trajectory planning and airspace utilization. DGGSs are a discretization of the Earth into hierarchical sets of highly regular grids, each of which represents a distinct region to which data may be assigned [37]. In this research, we referred to GeoSOT-3D theory to construct the basic spatial reference framework. The airspace is divided into regular 3D grids according to the GeoSOT-3D schema. The proposed air traffic highways in this research are constructed by 3D grids. Apart from UAV-related applications, GeoSOT-3D has been extensively investigated in remote sensing data management [38, 39], city component identification [40], trajectory data storage [41], and urban expansion monitoring [42].

**Fig 1:**
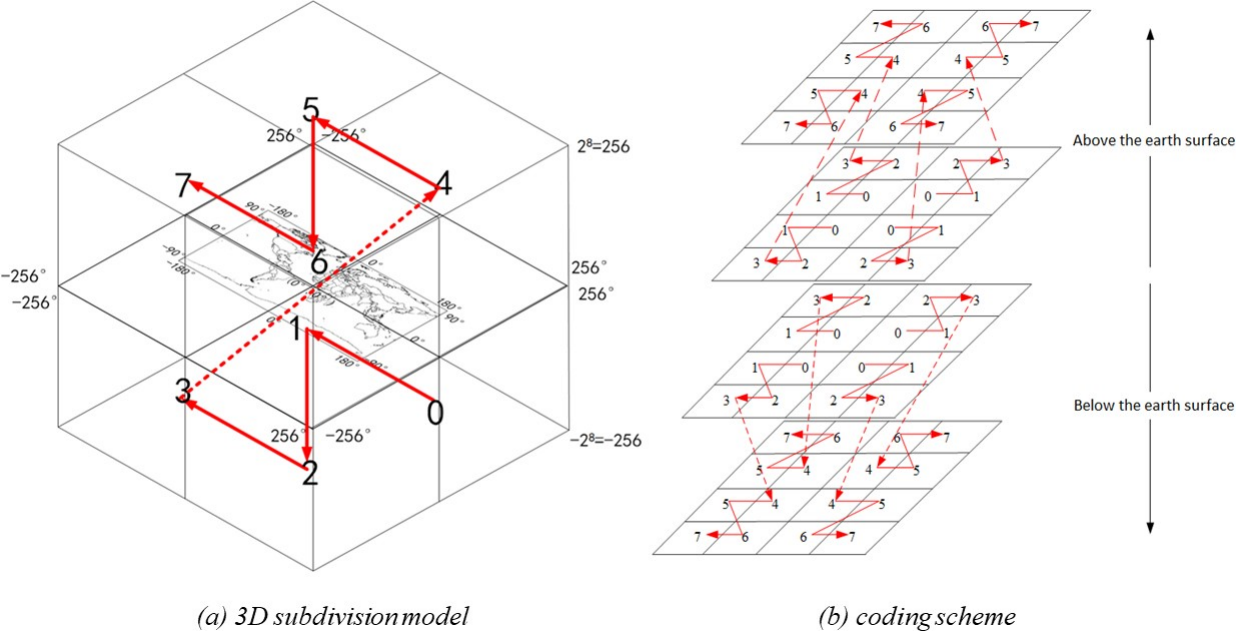
Structure of GeoSOT global subdivision grids. A dichotomy algorithm is applied in the three directions of longitude, latitude and altitude. The binary digit in the code is assigned 1 if the original data is higher than the mean value at the corresponding level; otherwise, the binary digit in the code is assigned 0. The 1st-level grid encoding order is shown in subfigure (a). The grids at each level below the 1st level should be encoded based on the upper-layer grid codes. Specifically, in the height direction, the encoding order extends from the lower level to the upper level, and the Z-order is adopted to continue encoding at the same level. The direction of Z-order coding is determined by the locations of the 1st-level grids. An example of the encoding order is shown in subfigure (b).

The GeoSOT-3D system proposed for this study is a universal and hierarchical three-dimensional geospatial reference system [43]. By subdividing the Earth via iterations (first, expanding the Earth (180°×360°) into 512°×512° grids; second, expanding each 1° into 64’; and last, expanding each 1’ into 64”), two-dimensional quadtree subdivisions at the degree, minute, and second levels are obtained. In addition, we extended the two-dimensional global subdivision framework into a third spatial dimension: elevation. The division of the elevation dimension is directly performed using a dichotomy. The entire range over which the elevation dimension is divided spans from the geocentric position to 50,000 km above sea level. The largest subdivision grid at the highest level (Level 0) of GeoSOT-3D can represent the entire Earth space, while the smallest subdivision grid at the lowest level (Level 32) can represent the centimeter scale. In this way, the GeoSOT-3D grid system not only possesses global multidimensional octree hierarchical characteristics but also maintains consistency with different types of applications. With an octree-like structure, GeoSOT-3D coding exhibits 3D components. The node index that branches out from the top level is determined by a formula that uses bits to characterize the longitude, latitude, and altitude within the former node’s space. The digit is either 0 or 1 at each level in one dimension; therefore, a digit that ranges in value from 0 to 7 can be applied to represent one code at each level by occupying 3 bits. The next (lower) level inherits the digit from the former digit, and an identical formulation is repeated, which is shown in Fig 1. The 3D subdivision model and coding scheme adopts the Z-sorting method, that is, a 3D coding cross-combination, to avoid the low query efficiency due to the high priority of a certain dimension when querying the spatial database.

The construction of GeoSOT-3D code is based on a binary system, which is in accordance with the storage structure of common computer systems and databases. The coding can be expressed by integers, which is suitable for all kinds of storage computing architectures and has high efficiency. Currently, the GeoSOT-3D grid has been employed in the intelligent construction project of UAVs in Cangshan, Fujian, China. Via grid modeling in the airspace, UAVs can achieve complex functions, such as delivery and intelligent path finding and obstacle avoidance in the park.

Referring to the level setting of China’s highway system, we constructed an air route system that contains four types of low-altitude air routes—national routes, provincial routes, county routes, and township air routes—to prepare for air flight at the national level, provincial level, county level and township level, respectively. Specifically, the air routes are constituted by grids at the same level. The selected grid level and related route properties are indicated in Table 1.

**Table 1:** Air route type setting.

Air route type	GeoSOT grid level	Size	Edge length
**National**	*15*	1’*1’	1.85 km
**Provincial**	*19*	4”*4”	128 m
**County**	*20*	2”*2”	64 m
**Township**	*21*	1”*1”	32 m

The GeoSOT grid level is selected to satisfy the needs of various types of air routes. Grids at each selected level are identical cubes. The edge length is not fixed because of variations introduced by the latitude and altitude of the grids. However, in the construction of UAV low-altitude air routes, variations can be disregarded because variations in the latitude and longitude of the UAV range are limited in actual applications.

**Fig 2:**
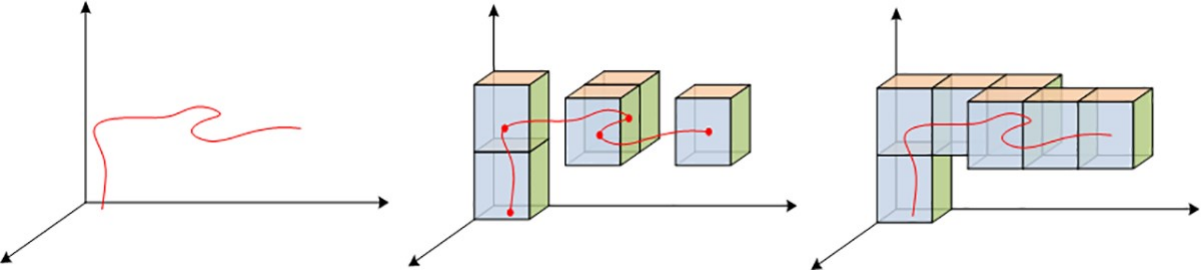
Construction procedure of one air route by grids.

In this research, we referred to the idea of DGGSs in attempting to design a framework of low-altitude air routes. The construction of the proposed method consists of three major steps: 1) identify the 3D grids according to the vertices along the route; 2) retain the successive identified grids if they are spatially adjacent; and 3) connect the successively identified grids, if they are not spatially adjacent, to algorithms such as the Bresenham algorithm [44, 45]. Fig 2 demonstrates a typical air route construction procedure.

**Fig 3:**
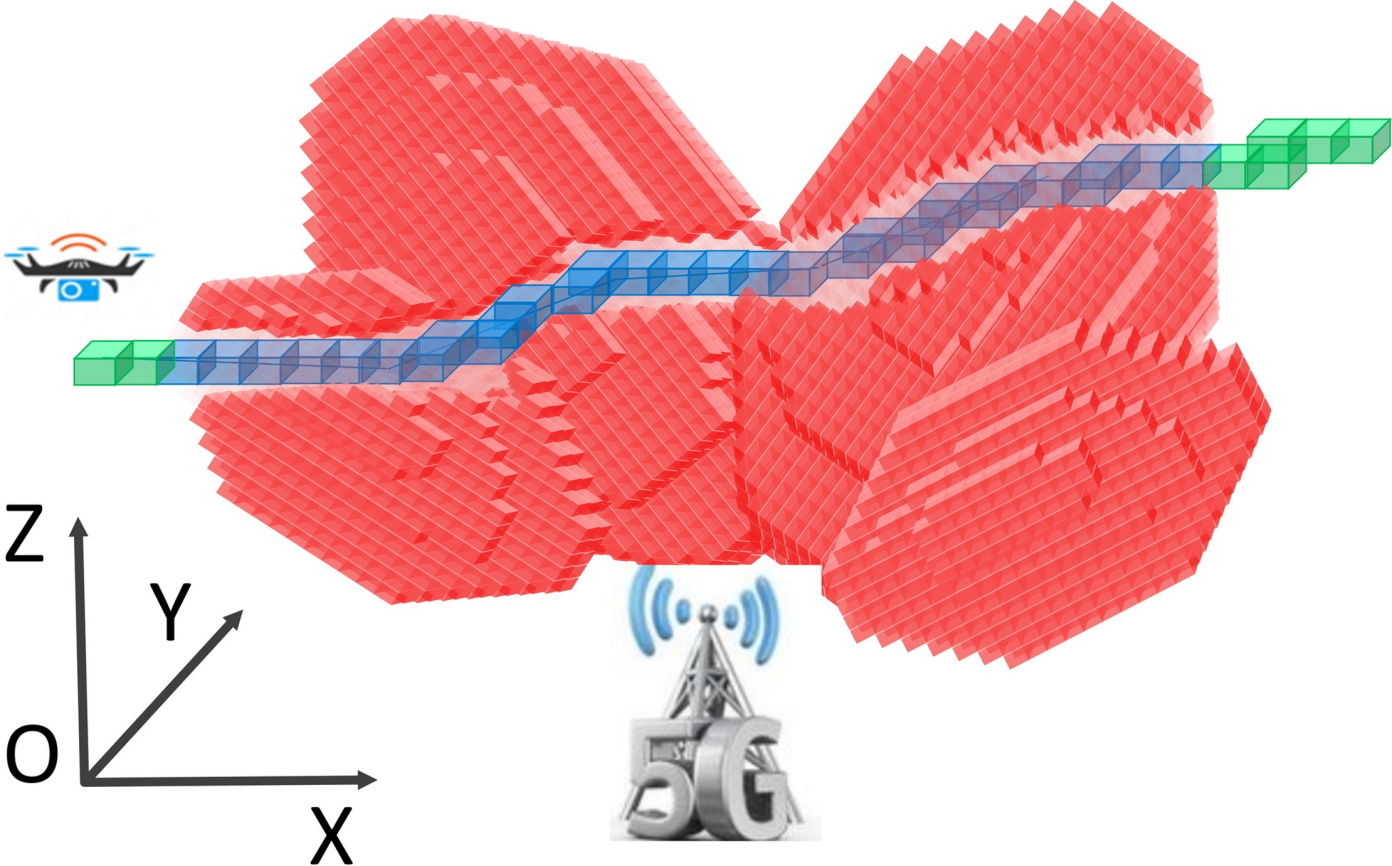
Schematic of 5G signal coverage along a UAV air route. The spatial coverage of the 5G signal is represented by massive regular grids. The size and position of each grid are defined according to the GeoSOT-3D coding schema. The air routes of UAVs are also constructed by consecutive grids. Blue grids demonstrate that the signal strength in the grids is strong enough to satisfy the flight requirements of UAVs. Green grids indicate that the 5G signal is weak, and there may be a safety risk when UAVs fly in these grids.

With the continuous development and wide application of fifth-generation (5G) communication technology, the flight of a low-altitude UAV needs 5G signal coverage along its route. Beamforming is an effective multiantenna technique that dynamically adjusts the antenna radiation pattern based on user location or even instantaneous channel state information (CSI) [46]. The preliminary studies in [14] demonstrated the promising gains of 3D beamforming over the conventional base station (BS) antenna configuration with a fixed radiation pattern [46, 47]. The potential combinations of UAVs and 5G technology highlight several aspects: charging efficiency [48]; UAV-to-UAV and satellite-to-UAV communications [49, 50]; security and privacy [51]; and the integration of networking, computing and caching [52, 53]. Massive 5G base stations and complex low-altitude public air routes pose a challenge to guaranteeing coverage. From the perspective of time and space, low granularity and massive data volume render exploiting 5G beamforming in UAV applications challenging. However, the construction of gridded air routes simplifies the problem. To ensure flight safety, the intensity and distribution of signals should be adjusted or the air route should be reconstructed, as indicated in Fig 3.

**Fig 4:**
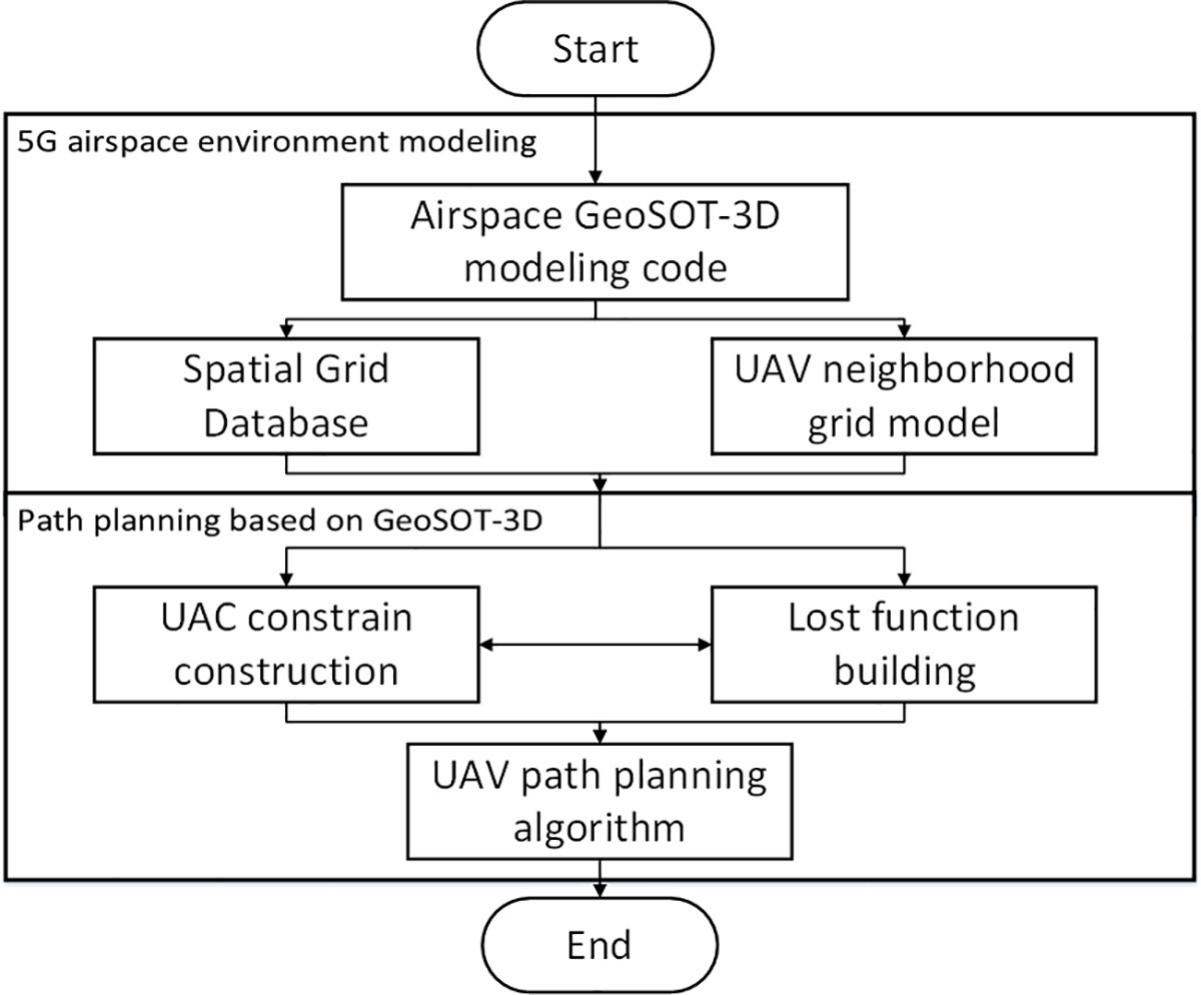
UAV trajectory planning process in 5G signal field environment.

In the 5G beamforming environment, the UAV can complete a flight to maximize the 5G coverage area under GeoSOT-3D airspace grid coding. The method employed for flight is based on the GeoSOT-3D trajectory planning algorithm. The objective of UAV path planning is to design an optimal feasible path in flight airspace given the UAV-related constraints and cost functions. Based on the global three-dimensional subdivision reference grid system, this paper establishes an environment model of the spatial grid inside the 5G signal field and develops a new path planning algorithm. When the UAV is flying within the 5G signal range, it is necessary to complete the 5G field grid environment modeling and track search. The environmental modeling phase requires that the airspace needs to be processed by GeoSOT-3D gridding, which causes the airspace to become multi-scale and unique-code. According to the results of the airspace spatio-temporal division, the track search phase can be based on the improved A* trajectory planning algorithm of the airspace grid, taking the UAV’s reachable neighborhood grid as the unit, by searching for the optimal neighborhood grid during flight and forming the most feasible flight path to ensure that the UAV can be as far as possible within the coverage of 5G beamforming. The UAV follows the flow shown in Fig 4.

## Results

**Fig 5:**
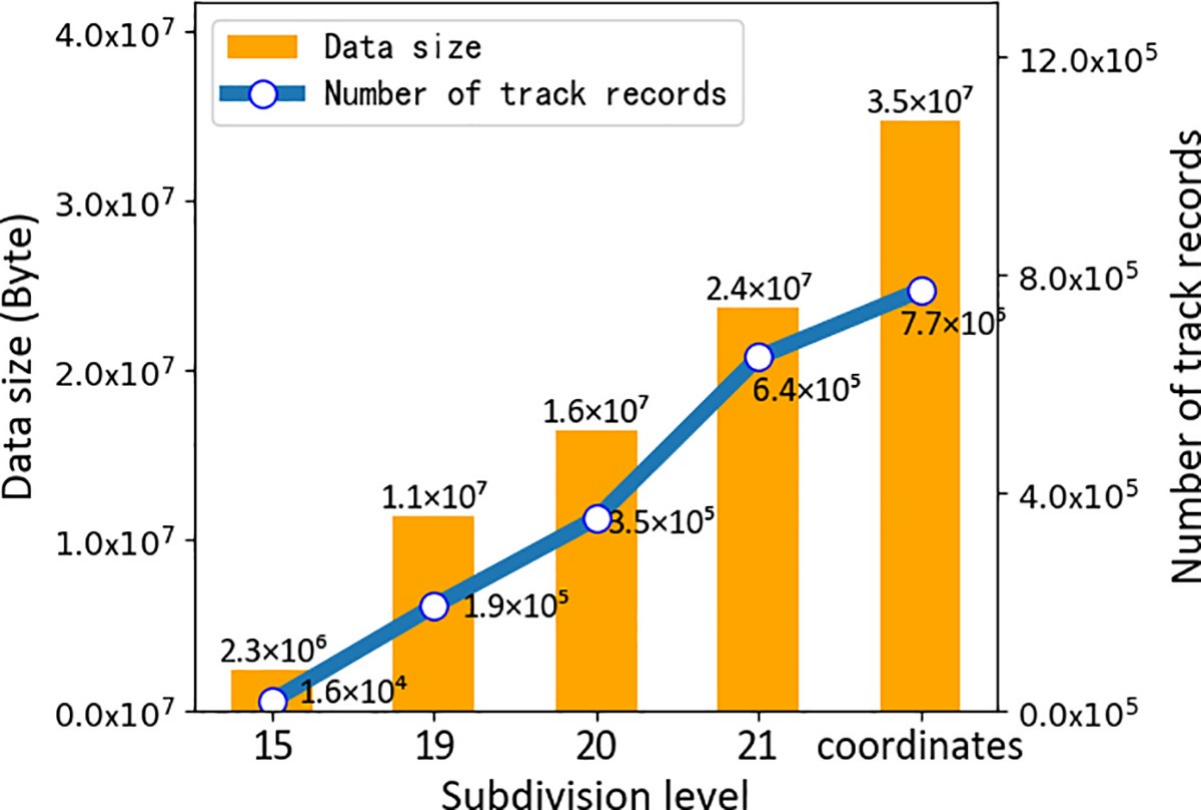
Data size and number of track records at different subdivision levels. Data size refers to the storage size of the airspace, 5G and UAV information under the same airspace using different levels of GeoSOT-3D grids and coordinates methods. The number of track records indicates the number of UAV tracks stored in the airspace.

We performed simulation experiments to verify the improvement in UAV management via the use of GeoSOT subdivision grids. The implementation of the grids changes the data storage schema. Previously, the spatial information of air routes and other objects was recorded by ObjectID, while in our design, a database with GeoSOT codes as the primary key was constructed, and both the air route and the object data were recorded in a nonsql database. We simulated the airspace environment in the latitude range of 44° and longitude range of 29.8° and added several actual track data to the airspace. After completing the airspace modeling, we compared the number and data size of stored track records using longitude and latitude and different grid levels. The data size and the amount of information recorded by the subdivision grids increase with the level, as shown in Fig 5, because a higher subdivision grid level generates a more detailed description of air routes and spatial objects and consumes more storage. If the data are not converted to grid representation, both the data size and the number of track records are larger than those after conversion. The experimental results show that the amount of data storage can be significantly reduced by subdivision. The data size using traditional expressions of latitude and longitude is approximately 1.5 times that using a 21-level grid, which belongs to a high grid level.

**Fig 6:**
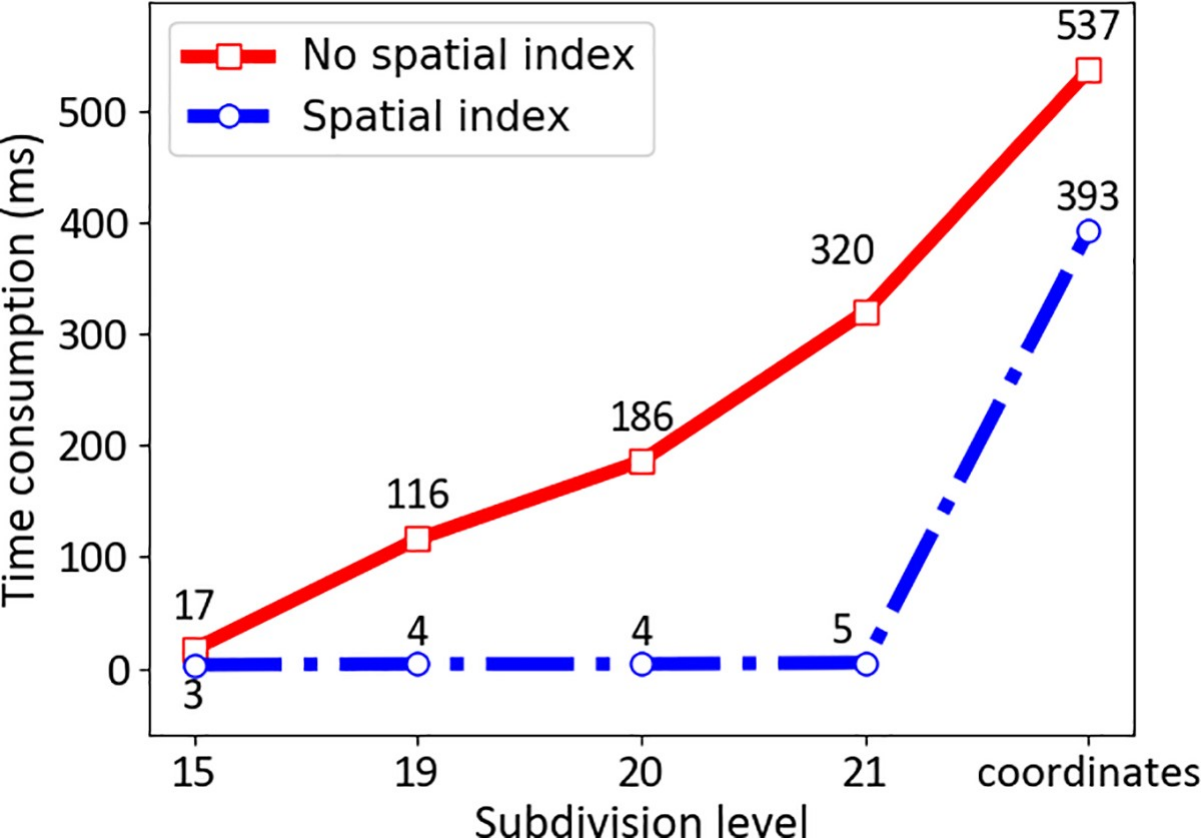
Time consumption for air route trafficability computation. Time consumption represents the average time it takes for the UAV to search its neighborhood with or without a large database supporting index.

We compute the time consumption for the air route trafficability, as indicated in Fig 6. The time consumption in the proposed methods with different levels is lower than that of the traditional method, i.e., computation by 3D coordinates. The time consumption by coordinates is approximately 1.5 times that of the subdivision grids at level 21. In addition, the incorporation of a spatial index contributes to decreasing the time consumption. At level 21, the time consumption with the spatial index is only 1.6% of the time consumption with no spatial index.

**Fig 7:**
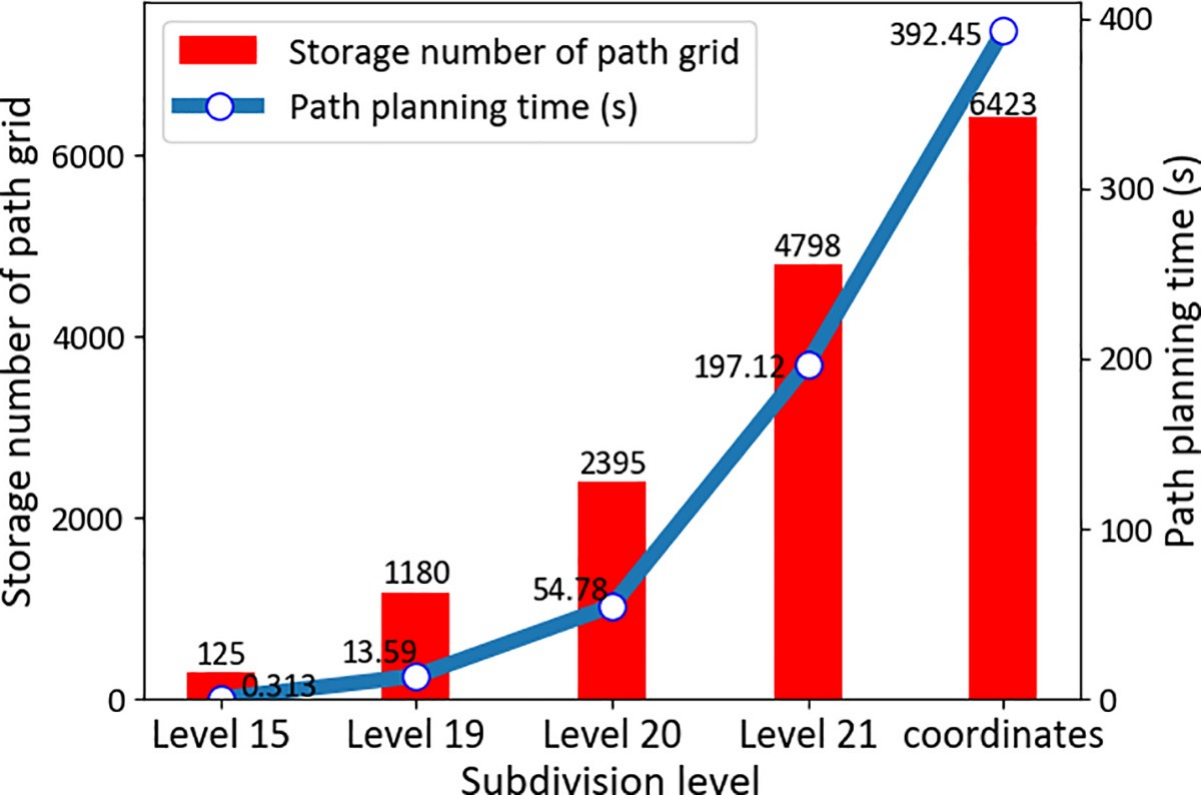
Time and storage consumption for path planning. In an identical airspace, with the same starting and ending points, this paper uses different environmental modeling methods to plan a UAV’s trajectory under the 5G field. The path planning time refers to the computing time required for a UAV to use the improved A* algorithm to obtain the optimal path. The storage number of the path grid refers to the number of trajectory grids generated in different environment modeling situations.

To solve the problem of UAV routes in the 5G environment, we utilized the GeoSOT-3D modeling method to grid 5G 3D beamforming and stored beam signal strength information in multilevel spatial grid data and applied it to the experiments in the intelligent construction project of UAVs in Cangshan, Fujian, China. In the construction of gridded air routes, when a UAV is in a low-altitude airspace, it will tend to fly in an area with a stronger 5G signal, and the track should never leave the coverage area of the 5G beam. In the experiment with a 5G signal airspace field, we simulated UAV flight at GeoSOT grid levels 19, 20, and 21 and 3D coordinates. The final path planning time and optimal path storage number are shown in Fig 7. The experimental results show that in low-altitude airspace, due to the large number of 5G beams and massive environmental data, the traditional longitude and latitude coordinates method leads to a large planning time and storage need. However, global subdivision grids accomplish the requirements of rapid UAV path design in a 5G signal beam field and can quickly complete UAV route construction in a 5G signal field at a large grid level.

In the airspace grid database, GeoSOT-3D could establish grid code databases of different levels and sizes according to actual needs to store airspace, UAV, and 5G information at the grid level that adapts to the current drone flight. In the same airspace, the storage size is half of that in the coordinates method. In terms of the consumption time of the neighborhood position, the GeoSOT-3D time is approximately 35% of that for the coordinates method with the database index, while the GeoSOT-3D time based on the general coordinates of the path planning time is 7 times the calculation time based on GeoSOT-3D path planning. It is easy to understand that the GeoSOT-3D spatio-temporal division grid, as a storage method of airspace expression during UAV flight, has great advantages compared with traditional longitude and latitude methods. The specific data comparison is shown in Table 2.

**Table 2:** Airspace performance comparison between coordinates and GeoSOT-3D at the 20th level.

	GeoSOT-3D (Level 20)	Coordinates	Proportion
**Data Size**	1.6×10^7^ (KB)	3.5×10^7^ (*KB*)	1:2.2
**Time consumption for trafficability computation (spatial index)**	186 (ms)	537 (ms)	1:2.9
**Path planning time**	54.78 (s)	392.45 (s)	1:7

## Discussion

In this research, to better address future complex low-altitude UAV management issues, we advocate the concept of gridded air route construction. Four types of gridded routes are specifically designed for UAVs based on the sizes of the grids. The selection of grids is based on the development level of UAVs now and in the near future. With an increase in UAV quantity, the improvement in communication tools, variations in the spatial environment and changes in related laws or regulations, the sizes of the grids are adjustable. In addition, the applications of grids in other fields enable the integration of information from multiple sources, which creates smart routes and further enhances UAV management.

The discretization of space by grids for improved management of spatial objects, such as UAVs, has gained attention and recognition in various domains. The design of gridding was mentioned in the ANSI UASSC in June 2020, which indicates that the industry considers this issue important. The essence of the subdivision process is the discretization of continuous space. Based on this framework, each grid corresponds to one unique code, which acts as a primary key in the spatial database, restoring spatial information within the grid.

UAV flight demands the support of 5G communication technology, which requires that the construction of UAV low-altitude air routes consider signal coverage. If we analyze the parts within the low-altitude air routes in detail to confirm the 5G signal coverage, a large amount of computational resources will be needed. In addition, this complex computation process can be time-consuming in a dynamic environment. The application of grids provides an alternative approach to simplify the process. After the initialization of the grids, the signal strength within each grid is recorded in the database. To determine whether a location is suitable for flight, we need to only check the occupancy and coverage of the grid that contains the location. In addition, it is easy to calculate the effect of enhancement and weakening among signal fields released by different base stations. In the dynamic environment, variations or changes in a signal field and the environment are reflected in the updated information restored in the database, which facilitates efficient positioning and computation.

## Conclusions

UAVs have essential roles in an increasing number of applications. However, there are no air traffic routes for managing and standardizing massive numbers of UAV flights. Existing management approaches to UAV flights include implementing registration of flight activity for supervision purposes, limiting the maximum flight height, setting different zones for different flight activities and prohibiting flights. Nevertheless, managing the utilization of low-altitude airspace by traditional methods is limited. In addition, as the number of UAVs increases rapidly in the future, traditional methods will become increasingly time-consuming and inflexible. In this research, borrowing the idea from highway construction and design in ground transportation, we proposed a new air traffic management method for UAVs. In contrast to previous related research on constructing air routes with corridors, the proposed low-altitude air routes are constructed based on global subdivision theory. Three-dimensional grids at different levels are the basic elements for constructing air routes and other spatial objects. We can standardize the UAV-related data, and the codes that correspond to the grids can be acquired as the primary key in the spatial database, which enables high query efficiency. We designed four types of low-altitude air routes from grids, which correspond to grid sizes of 1.85 km, 128 m, 64 m and 32 m.

In addition, considering the widespread use of 5G technology and the tight coupling of 5G technology and UAVs, how to combine UAV applications and 5G technology remains to be solved. The utilization of subdivision grids transforms the complex spatial computation problem into a query process in the spatial database, which provides a new approach to UAV management in the 5G era.

We conducted three types of experiments to verify the feasibility of constructing this type of air traffic highway system. First, we compared the number and data size of stored track records using longitude and latitude and different grid levels. The amount of data storage can be significantly reduced by subdivision. For example, the data size using traditional expressions of latitude and longitude is approximately 1.5 times that using a 21-level grid. Second, we computed the time consumption for air route trafficability. The time consumption for the proposed methods with different levels is lower than that of the traditional method, i.e., computation by 3D coordinates. The time consumption by coordinates is approximately 1.5 times that of subdivision grids at level 21. Third, we simulated UAV flight at GeoSOT grid levels 19, 20, and 21 and 3D coordinates. The experimental results indicate that in the 5G environment, gridded airspace can effectively improve the efficiency of UAV trajectory planning and reduce the size of airspace environment information storage. It is promising, given the increasing number of UAVs in the future, that gridded highways have the potential to provide a foundation for various UAV applications.
